# Esophageal retention cyst: Esophagogastric junction outflow obstruction (EGJOO) as a potential etiology and management with endoscopic mucosal resection (EMR)

**DOI:** 10.1016/j.ijscr.2022.107194

**Published:** 2022-05-11

**Authors:** Nisarg Mehta, Shahin Ayazi, Michael Landau, Sven Eriksson, Blair A. Jobe

**Affiliations:** a Esophageal Institute, Department of Surgery, Allegheny Health Network, Pittsburgh, PA, United States; b Department of Surgery, Drexel University, Philadelphia, PA, United States; c Department of Pathology, Allegheny Health Network, Pittsburgh, PA, United States

**Keywords:** Esophageal retention cyst, Esophageal mucocele, Esophagogastric junction outflow obstruction (EJGOO), Jackhammer esophagus, High resolution manometry, Dysphagia, Endoscopic mucosal resection (EMR)

## Abstract

**Introduction and importance:**

Esophageal retention cysts are acquired cysts with no known etiology. They are characterized by dilation of the submucosal glands. Symptomatic cysts are traditionally managed by surgical resection.

**Case presentation:**

We present a case of progressive dysphagia and chest pain secondary to esophageal retention cysts in the mid and distal esophagus with associated esophagogastric junction outflow obstruction (EGJOO) and jackhammer esophagus on high resolution manometry (HRM). The patient underwent staged endoscopic mucosal resection (EMR) with subsequent improvement in her symptoms. However, EGJOO persisted after resection, suggesting it was the primary pathology and not a consequence of the obstruction from the cysts.

**Clinical discussion:**

Esophageal retention cysts are rarely reported in the literature with most descriptions coming from incidental post-mortems. The presented case suggests EGJOO as a potential etiology of retention cysts. The proposed mechanism is that a significant rise in esophageal intraluminal pressure creates a state of stasis in the esophagus, ideal for the development of these cysts. Symptomatic or malignant retention cysts should be resected. We demonstrate the feasibility of EMR as an alternative to surgical resection.

**Conclusion:**

Esophageal retention cyst is a rare entity, which may arise as a result of EGJOO. The natural history and malignant potential of these cysts are unknown, and no formal guidelines have been established for follow-up for patients with asymptomatic retention cysts. Endoscopic mucosal resection can be used to successfully manage these cysts.

## Highlights

• Esophageal retention cysts are rare and characterized by dilation of the submucosal glands.
• The esophageal retention cyst is an acquired cyst. The exact etiology is unknown, but several conditions have been proposed as the potential explanation.
• This increase in the intraluminal pressure from esophagogastric junction outflow obstruction (EGJOO) may lead to obstruction of the submucosal duct and formation of the retention cysts.
• The natural history and malignant potential of these cysts are unknown, and no formal guidelines have been established for follow-up of patients with asymptomatic retention cysts.
• Endoscopic mucosal resection can be used to manage these cysts safely and effectively.

## 1 Background

Esophageal retention cysts are characterized by dilation of the submucosal glands with intact overlying squamous epithelium [1]. The cysts are presumed to arise from obstruction of the excretory ducts of these glands, but the exact etiology of their formation is unknown [1]. The most common associated symptom with retention cysts is dysphagia, although most are asymptomatic [2]. Here we present a rare case of progressive dysphagia secondary to esophageal retention cysts with associated esophagogastric junction outflow obstruction (EGJOO) and jackhammer esophagus on high resolution manometry (HRM). To our knowledge this is the first reported case of EGJOO that resulted in the formation of esophageal retention cysts. We discuss the endoscopic management of this patient and explore how this case impacts our understanding of esophageal retention cysts. This work has been reported in line with the SCARE 2020 criteria [3].

## 2 Case presentation

**Fig. 1:**
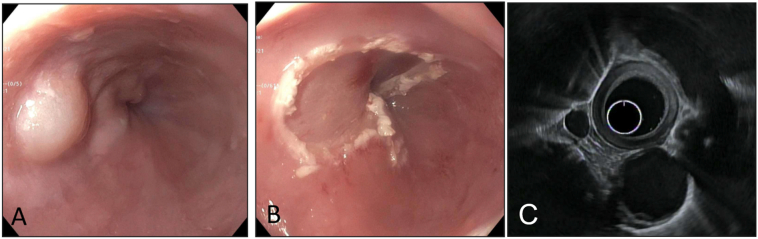
Preoperative EGD, post EMR EGD and EUS images: A) Endoscopic view of one of the retention cysts in the mid esophagus, note the intact mucosa. B) Cyst after resection using band-ligation assisted EMR. C) Endoscopic sonographic image of one of the cysts, showing a well-defined submucosal anechoic structure. (EGD: Esophagogastroduodenoscopy, EMR: endoscopic mucosal resection, EUS: Endoscopic ultrasonography).

**Fig. 2:**
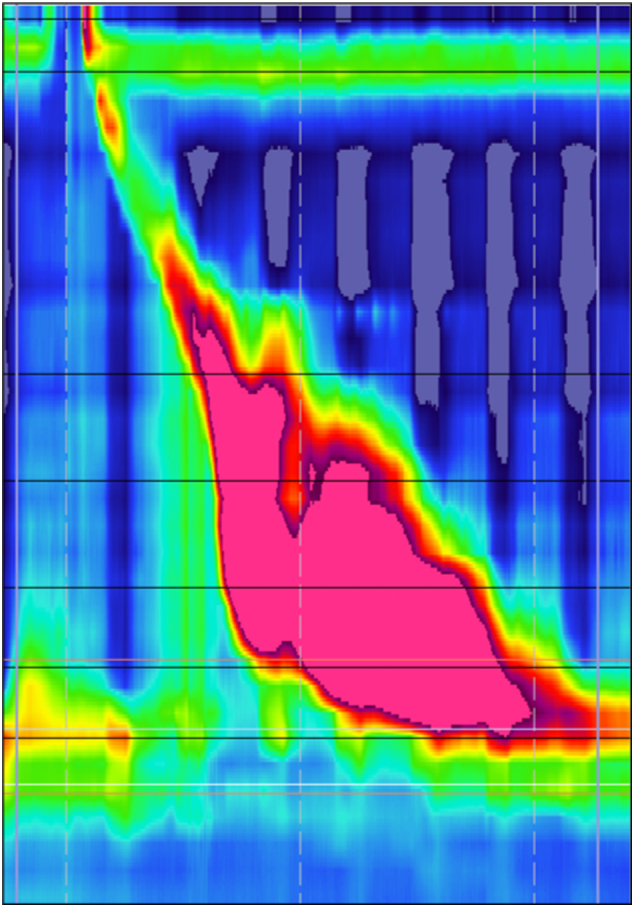
A sample contraction from HRM topographic plot of the patient. This test showed elevated LES resting pressure of 86.7 mmHg and elevated IRP of 31.6 mmHg consistent with diagnosis of EGJOO. Patient was also found to have an elevated mean DCI of 14,665.9 mmHg·cm·s with all contractions with DCI > 8000 mmHg·cm·s consistent with the diagnosis of jackhammer esophagus. (HRM: high resolution manometry, IRP: integrated relaxation pressure. EGJOO: esophagogastric junction outflow obstruction, DCI: distal contractile integral).

**Fig. 3:**
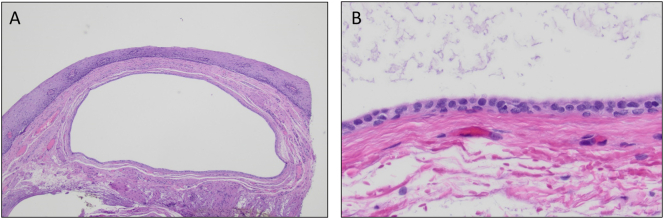
Histologic review of resected specimen. A) cyst involving the esophageal submucosal ducts (H&E, orig. mag. ×40). B) High power image of the cyst lining epithelium composed of bland cuboidal to columnar cells. (H&E, orig. mag. ×400).

Patient is a 58 years-old female with history of gastroesophageal reflux disease (GERD), obesity (BMI: 38.7), asthma, diabetes mellitus and hypertension. She had been experiencing dysphagia and chest discomfort for more than 10 years; worsening of her chest pain prompted her to visit the emergency room. Following a negative cardiac work-up, she was referred to a gastroenterologist for an endoscopic evaluation. The esophagogastroduodenoscopy (EGD) report described a few medium-sized blebs in the lower third of the esophagus. The patient was then referred to our tertiary care center for further evaluation and management. To further characterize the lesions, an EGD with endoscopic ultrasound (EUS) was performed, which re-demonstrated several cystic lesions in the distal and middle esophagus, and showed several well defined anechoic submucosal lesions (Fig. 1). High resolution manometry (HRM) was then performed which showed a hypertensive lower esophageal sphincter (LES) with an elevated resting pressure of 86.7 mmHg and elevated integrated relaxation pressure (IRP) of 31.6 mmHg consistent with the diagnosis of EGJOO (Fig. 2). In addition, the mean distal contractile integral (DCI) was elevated at 14665.9 mmHg·cm·s and all contractions had a DCI > 8000 mmHg·cm·s, consistent with the diagnosis of jackhammer esophagus. The decision was made to perform endoscopic mucosal resection (EMR) of these cysts in a staged manner (Fig. 1). Pathologic evaluation of the lesions showed benign squamous mucosa with cystic dilation of the submucosal duct consistent with esophageal retention cyst (Fig. 3). Of note, pathologic examination of tissue obtained from non-cystic parts of the esophagus also showed microscopic submucosal retention cysts.

**Fig. 4:**
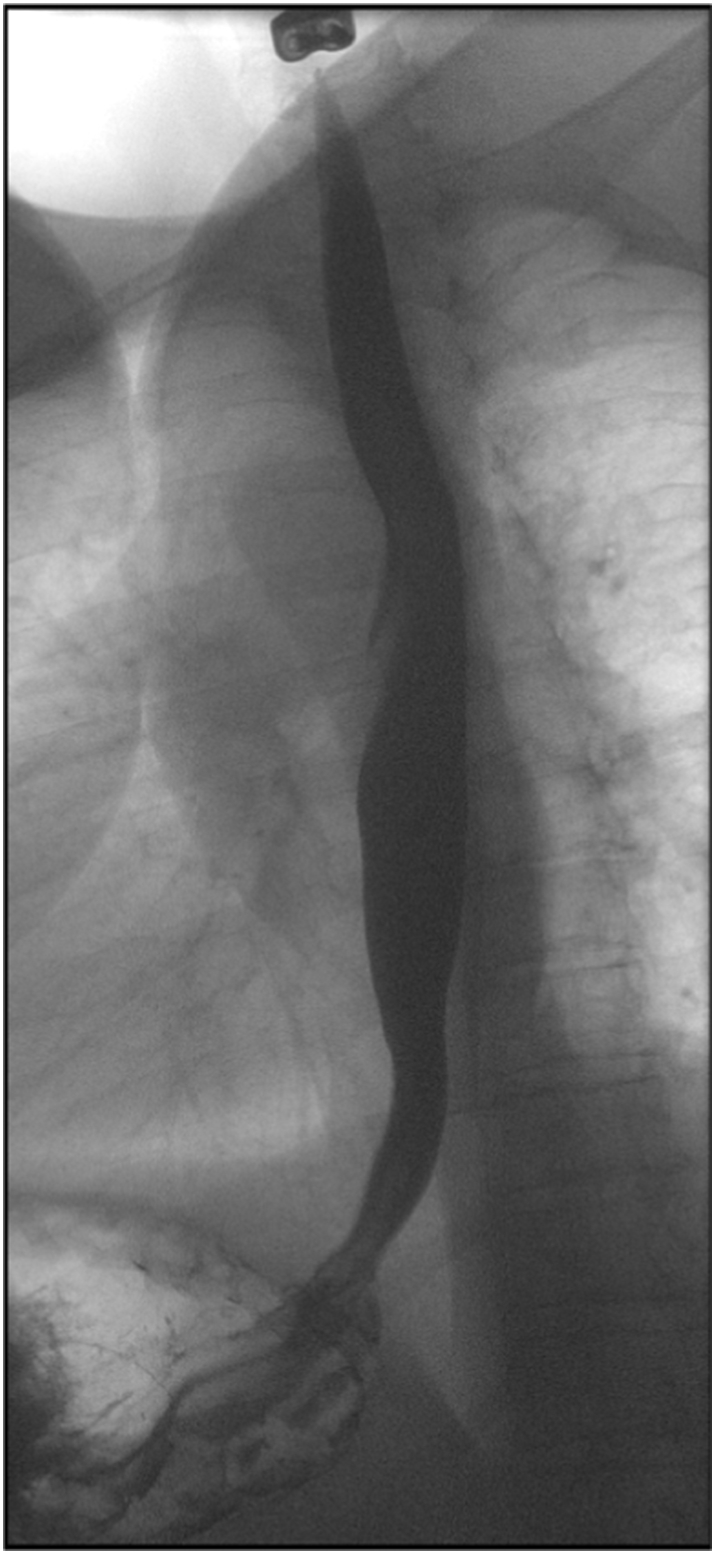
Esophagram obtained after endoscopic resections showing no cystic lesion in the esophagus, but ingested 13 mm barium tablet was retained above esophagogastric junction for more than 3 min.

Following EMR, the patient reported significant symptomatic improvement, and post-operative esophagram showed no further cystic lesion in the esophagus. However, the ingested 13 mm barium tablet was retained above EGJ for more than 3 min, concerning for persistent outflow resistance at the EGJ (Fig. 4). HRM was repeated following resection and showed an LES resting pressure of 65.6 mmHg with IRP of 36.7 mmHg and mean DCI of 11,480.7 with all contractions with DCI > 8000 mmHg·cm·s. The patient was enrolled in a weight management program and was being counseled for bariatric surgery prior to presenting to us. Since her esophageal dysmotility persisted even after eliminating mechanical obstruction from the cysts and given the unknown natural history or malignant potential of esophageal retention cysts, she was advised to not proceed with a bariatric surgical intervention. The altered foregut anatomy following a bariatric procedure could pose a challenge if she requires an esophagectomy in the future. The patient is currently considering a surgical or endoscopic myotomy to address her EGJOO and jackhammer esophagus.

## 3 Discussion

The esophageal retention cyst is a rare type of acquired cyst that was first reported by Kuhne in 1899 [4]. Since that time, there have been scarce reports of these lesions [1], [2], [5], [6], [7], [8], [9], [10]. The majority of descriptions come from incidental findings on autopsy, and use a variety of terms including: ‘mucocele’, ‘esophageal cystica’, ‘esophageal submucosal gland cyst’, and ‘retention cyst’ [1], [2], [4]. Found throughout the esophagus, but preferentially involving the distal third, retention cysts can appear as individual or multiple lesions [6]. The size of these cysts have been reported to range from sub-centimeter to up to 15 cm, with the larger cysts more frequently associated with symptoms [7]. Dysphagia is the most common symptom with some patients also complaining of abdominal pain, chest pain and weight loss [1], [8]. Similarly, the patient in this case report complained of progressive dysphagia and chest pain and was found to have multiple retention cysts distributed in the middle and distal third of esophagus.

The exact etiology of the retention cyst is not known, but the cellular structure of the esophagus provides clues as to how these cysts may form. The submucosal layer of the esophagus is lined with tubulo-alveolar glands which drain into the lumen through cuboidal and squamous cell lined ducts [2]. Blockage of these ducts leads to an accumulation of mucous in the tubulo-alveolar glands, causing enlargement, and eventual development of a retention cyst [2]. Proposed etiologies of this submucosal duct obstruction have included inflammation of the esophageal mucosa, GERD, abnormal submucosal gland function, basal cell hyperplasia at the duct orifice, and esophageal dysmotility [2], [5].

Following the initial endoscopic and manometric examination of the patient in this case, the assumption was that her symptoms, EGJOO and jackhammer esophagus were all disturbances caused by the retention cysts. The expectation following successful resection was that her symptoms would improve and her manometry would normalize. However, her post-operative manometry was essentially unchanged, despite improvement in her symptoms. This finding suggests that her retention cysts were not the primary pathology, but a result of EGJOO.

Based on the findings of this case we propose an evidence-based mechanism as to how EGJOO might have resulted in this patient's retention cyst formation. In order to overcome the elevated resistance at the EGJ, a compensatory contractile force was gradually formed in the esophageal body, eventually leading to jackhammer esophagus. A similar compensatory force has been suggested by animal studies, demonstrating that distal obstruction results in an increase in the esophageal smooth muscle contractile force [11]. Additionally, a similar augmentation of the contractile amplitude and increase in DCI has been reported following the increase in EGJ resistance after magnetic sphincter augmentation [12]. As this compensatory force developed, the intraluminal pressure increased and a state of esophageal stasis was created. Secretory build-up in this state of stasis blocked the submucosal ducts and led to retention cyst formation [13].

It has even been suggested that retention cysts may be a precursor to submucosal gland adenocarcinoma [14]. The evidence for this relationship, however, is only circumstantial. The argument being that similar demographic, distributional and histological features suggest a common etiology [14]. However, esophageal adenocarcinoma can arise from tissue that has undergone metaplasia, as seen with GERD and Barrett's esophagus. In patients with esophageal retention cysts, metaplasia has been described secondary to chronic inflammation within the cyst, which may be able to progress to dysplastic or malignant tissue [13]. The likelihood of this malignant transformation is unknown, but it has been suggested in the literature [6]. In the present case, tissue specimens taken from healthy appearing esophageal mucosa showed dilated submucosal ducts concerning for early retention cyst formation.

Historically retention cysts have been managed with esophagectomy or resection via thoracotomy or video assisted thoracoscopy [5], [6], [7], [8]. Percutaneous drainage of the retention cysts has been attempted in the pediatric population [8]. Successful CT guided drainage and alcohol induced mucosal ablation of cysts that were not located near vital structures have also been reported in the literature [6]. However, resection remains the most definitive treatment. With the advancement of endoscopic technique, EMR is now being used as the organ-sparing modality for treatment of benign and malignant esophageal lesions [15]. This approach significantly decreases the post-operative morbidity [15]. The present report demonstrates the feasibility of EMR for the management of retention cysts. No formal guidelines have been established for follow-up of asymptomatic retention cysts.

## 4 Conclusion

Esophageal retention cyst is a rare entity with an unknown etiology and a potential for significant morbidity. The patient described here presented with worsening dysphagia and chest discomfort and was found to have retention cysts on EGD with EUS and EGJOO and jackhammer esophagus on HRM. Following resection, symptoms improved, but motility did not. This finding suggests that EGJOO was the primary pathology, which led to an extreme rise in the intraluminal pressure, subsequent obstruction of the submucosal ducts and formation of esophageal retention cysts. Symptomatic retention cysts should be treated to resolve the symptoms and avoid potential complications. In the past, esophagectomy was considered the standard treatment, but this report shows that endoscopic mucosal resection can be used in management of retention cysts.

## Funding

Not applicable.

## Ethical approval

Not applicable.

## Consent

No identifying characteristics/features are noted throughout the case report. Written informed consent was obtained from the patient for this case report and accompanying images. A copy of the written consent is available for review by the Editor-in-Chief of this journal on request. Patients in our institution provide consent to allow use of their deidentified clinical information for research purpose.

## Author contribution

Equal contribution by all authors.

## Research registration number

Not applicable.

## Guarantor

Shahin Ayazi, MD.

## Provenance of peer review

Not commissioned, externally peer-reviewed.

## Declaration of competing interest

The authors have no relevant conflict of interest to disclose.
